# Multi-Locus GWAS of Quality Traits in Bread Wheat: Mining More Candidate Genes and Possible Regulatory Network

**DOI:** 10.3389/fpls.2020.01091

**Published:** 2020-07-31

**Authors:** Yang Yang, Yongmao Chai, Xuan Zhang, Shan Lu, Zhangchen Zhao, Di Wei, Liang Chen, Yin-Gang Hu

**Affiliations:** ^1^ State Key Laboratory of Crop Stress Biology for Arid Areas, College of Agronomy, Northwest A&F University, Yangling, China; ^2^ Institute of Water Saving Agriculture in Arid Regions of China, Northwest A&F University, Yangling, China

**Keywords:** bread wheat, quality traits, multi-locus Genome-Wide Association Study, quantitative trait nucleotides, regulatory network, candidate gene

## Abstract

In wheat breeding, improved quality traits, including grain quality and dough rheological properties, have long been a critical goal. To understand the genetic basis of key quality traits of wheat, two single-locus and five multi-locus GWAS models were performed for six grain quality traits and three dough rheological properties based on 19, 254 SNPs in 267 bread wheat accessions. As a result, 299 quantitative trait nucleotides (QTNs) within 105 regions were identified to be associated with these quality traits in four environments. Of which, 40 core QTN regions were stably detected in at least three environments, 19 of which were novel. Compared with the previous studies, these novel QTN regions explained smaller phenotypic variation, which verified the advantages of the multi-locus GWAS models in detecting important small effect QTNs associated with complex traits. After characterization of the function and expression in-depth, 67 core candidate genes involved in protein/sugar synthesis, histone modification and the regulation of transcription factor were observed to be associated with the formation of grain quality, which showed that multi-level regulations influenced wheat grain quality. Finally, a preliminary network of gene regulation that may affect wheat quality formation was inferred. This study verified the power and reliability of multi-locus GWAS methods in wheat quality trait research, and increased the understanding of wheat quality formation mechanisms. The detected QTN regions and candidate genes in this study could be further used for gene cloning and marker-assisted selection in high-quality breeding of bread wheat.

## Introduction

Bread wheat (*Triticum aestivum* L.) is one of the most important food crops worldwide, and is the third largest cereal crop in the world, just behind rice and corn (Asseng et al., 2011). About 20% of the energy, protein, and dietary fiber consumed by humans is provided by bread wheat (Ling et al., 2013). Another important objective in wheat breeding has long been to improve the quality traits, besides increasing yield (Nelson et al., 2006; Jin et al., 2016). Due to the importance of essential ingredients such as protein and starch in bread making, end-product quality, nutritional value, and economic impact, wheat quality breeding mainly focused on improving these basic ingredients (Suchy et al., 2007). Additionally, several physical, chemical, and rheological properties have to be determined to predict the quality of flour and dough (Reese et al., 2007; Suchy et al., 2007).

Among the quality traits, grain protein content (GPC) has received special attentions as a conventional indicator for measuring the nutritional value of food (Zhao et al., 2010). According to the solubility of protein components in different solvents, wheat protein can be divided into gliadin, glutenin, albumin and globulin (Singh and Skerritt, 2001). Among them, gliadin and glutenin are the main storage proteins of wheat, and the main constituents of wet gluten. Their content and composition affect the viscoelasticity and baking quality of wheat dough (Torbica et al., 2007). Several studies indicate that GPC and wet gluten content (WGC) are controlled by multiple genes, and some quantitative trait loci (QTLs) or genes are reported (Sun et al., 2010; Conti et al., 2011; Li et al., 2012; Maphosa et al., 2013). It has been confirmed that the *GPC* gene that regulates GPC is on the short arm of the group 6 chromosomes, and the subunit genes (*Glu-1*, *Glu-A1*, *Glu-B1* and *Glu-D1*) of high molecular weight (HMW) and the subunit genes (*Glu-A3*, *Glu-B3* and *Glu-D3*) of low molecular weight (LMW) genes that control WGC are on the long and short arms of the group 1 chromosomes, respectively (Uauy et al., 2006; Plessis et al., 2013). Furthermore, some QTLs of GPC and WGC were reported on all the 21 chromosomes of wheat (Zhang et al., 2008; Zhao et al., 2010; Bogard et al., 2012). In the SDS-sedimentation test, mixing flour with lactic acid caused the expansion and sedimentation of gluten, and high-quality and high-strength gluten would have a high SDS-sedimentation value (SV) (Peña, 2002). Therefore, SV can be used as an essential indicator for detecting the quality of gluten. Starch is mainly composed of two kinds of glucose polymers, amylose and amylopectin, and its content and composition affect the gelatinization characteristics, which directly determine the cooking quality (Toyokawa et al., 1989). Previous studies have confirmed that *waxy* genes encoding granule-bound starch synthase I (GBSS I) to control amylose synthesis in wheat, were mainly distributed on chromosomes 7AS, 4AL and 7DS (Ainsworth et al., 1993; Nakamura et al., 1995). Amylopectin synthesis is more complex than amylose and it mainly related to soluble starch synthase (SSS) including SS I, SS II and SS III. SS I and SS II are encoded by six genes on chromosomes 7AS, 7BS and 7DS, respectively, while SS III are encoded by two genes on chromosomes 1A and 1D (Nakamura et al., 2002; Yamamori and Endo, 1996). Also, multiple QTLs associated with total starch content (TSC) were found on wheat chromosomes 1A, 1D, 2A, 2D, 7A, 7B, 7D (Mccartney et al., 2006; Sun et al., 2009).

Wheat varieties with good grain test weight (GTW) usually have higher flour yield (FY), which is very important for millers (Gwirtz et al., 2006). QTLs related to GTW have been reported on all chromosomes except 1A and 6D (Narasimhamoorthy et al., 2006; Sun et al., 2010; Carter et al., 2012; Simons et al., 2012). Loci controlling FY have been determined on chromosomes 1A, 1B, 2A, 3B, 4A, 4B, 4D, 5A, 5B, 5D, 6A, 6D and 7A (Campbell et al., 2001; Kuchel et al., 2006; Nelson et al., 2006; Raman et al., 2009; Carter et al., 2012; Simons et al., 2012; Maphosa et al., 2013).

Dough rheological properties are the comprehensive performance of the flexibility and viscoelasticity of the dough. They are important indicators of wheat flour quality, and determine the final quality of bread, steamed bread, noodles and other wheat foods (Tsilo et al., 2013). The processing quality, especially the baking quality, of the final product of wheat flour is affected by three dough rheological properties, including dough water absorption (DWA), dough development time (DDT) and dough stability time (DST) (Spies, 1999; Zhu et al., 2001). In the past, wheat breeders mainly focused on the relationship between dough rheological properties, food processing quality and wheat flour quality, but few on the genetic basis of dough rheological properties (Tsilo et al., 2013). In a few previous studies, these three major traits were mapped on multiple chromosomes, such as DWA (1A, 1B, 2B, 4B, 4D, 5AL and 6B), DDT (1A, 1B, 1D, 7D), and DST (1A, 1B, 1D, 5D) (Kuchel et al., 2006; Mccartney et al., 2006; Ma et al., 2007; Li et al., 2013; Tsilo et al., 2013).

While these preliminary studies have strengthened our understanding of the genetic basis for wheat quality traits, it is not sufficient to use these observations to improve the quality of the wheat to boost the dough rheological properties, taking account of the multiple genetic regulation of whole wheat genome. Recently, as the development of DNA sequencing, Genome-Wide Association Study (GWAS) has become a powerful tool for analyzing the genetic basis of complex traits controlled by multiple genes. It has been applied to QTL and gene mapping studies in many species, such as rice, barley, maize, wheat (Huang et al., 2012; Yang et al., 2014; Fan et al., 2016; Wu et al., 2016; Guo et al., 2017). Classical GWAS mainly includes two models, the general linear model (GLM) and the mixed linear model (MLM). The MLM model is widely used because of its effective control of false- positive locus (Zhang et al., 2005; Yu et al., 2006). However, as a traditional single-locus GWAS, MLM is usually difficult to identify important loci with small effect due to the overly conservative Bonferroni correction (*p* = 0.05/*m_e_*, where *m_e_* is the number of effective markers) (Wang et al., 2016). To improve this disadvantage, several multi-locus GWAS methods, including mrMLM, FASTmrEMMA, FASTmrMLM, pLARmEB, and pKWmEB, have been developed. Good results have been obtained in QTL identification of complex traits in many species, such as free amino acid levels in wheat, photosynthetic traits in soybean, and agronomic traits in barley (Wang et al., 2016; Wen et al., 2017; Zhang et al., 2017; Hu et al., 2018; Lü et al., 2018; Peng et al., 2018; Ren et al., 2018; Tamba and Zhang, 2018). These multi-locus GWAS methods do not require strict Bonferroni correction, so in addition to improving the power and accuracy of GWAS, they can also identify the small-effect quantitative trait nucleotides (QTNs).

The conventional methods of measuring wheat quality include several kinds of professional tools for physical and chemical analysis. These are expensive, labor-intensive, time-consuming and grain consuming tools that make the selection of quality traits in the early generation more complicated for wheat breeders. With the improvement on measurement throughout, speed and accuracy by Near-infrared reflectance spectroscopy (NIRS) analyzer, NIRS has been widely used in the non-destructive analysis of grain components, such as grain protein content, oil, fatty acids, dietary fiber, moisture, seed physical traits (Cen and He, 2007). Compared with traditional laboratory methods, the NIRS methods have the great advantages of non-destructive, less time, low cost, and fast speed, and have been used for rapid phenotyping of quality traits in maize, soybean, rice, barley, oat and triticale (Delwiche et al., 1996; Windham et al., 1997; Fox et al., 2011; Tarr et al., 2012; Han et al., 2017; Xu et al., 2019).

In this study, to understand the genetic basis of the formation of wheat quality, six grain quality traits and three dough rheological properties of 267 wheat accessions were estimated by NIRS analyzer in three years’ environments, the wheat accessions were genotyped by 34,043 high-quality SNPs of the Axiom™ Wheat Breeder’s Genotyping Array (35K), and then GWAS were conducted for the above nine quality traits. To compare the differences between different methods and to find more reliable QTNs, two traditional single-locus (GLM and MLM) and five multi-locus GWAS methods (mrMLM, FASTmrEMMA, FASTmrMLM, pLARmEB and pKWmEB) were applied in this study.

The objectives of this study were to: (a) estimate the genetic variance and heritability of six grain quality traits and three dough rheological properties in multiple environments, and explore the correlations between these two types of quality traits; (b) detect QTNs associated with two types of quality traits and investigate the co-effect QTNs; (c) compare the detected QTNs with the previous studies, and compare the detection efficiency of single-locus and multi-locus GWAS methods; (d) mine the candidate genes in QTN regions and understand the regulation network of wheat grain quality. This study will provide more complete and accurate information for further gene cloning, and marker-assisted selection in wheat high-quality breeding.

## Materials and Methods

### Plant Materials

To ensure a broadly representative sampling of Chinese winter wheat, a total of 267 wheat accessions containing seven foreign materials and 260 accessions from five major winter production regions in China were used (**Supplementary Table S1**). All materials were grown during three winter cropping seasons (October to June of 2016–2017, 2017–2018 and 2018–2019) on the experimental farm of the Institute of Water Saving Agriculture in Arid Areas of China, Northwest A&F University, Yangling, Shaanxi, China (34°7’N, 108°4’E). Field trials were conducted in randomized complete blocks with three replicates, each genotype was planted in three rows 2.0 m in length, with 25 cm between rows and 3.3 cm between plants. The field experiment followed the standard local agronomic wheat management practice. Compound fertilizer (N:P_2_O_5_:K_2_O ratio of 20:18:5) of 750 kg/hm^2^ was applied before planting, and one supplemental irrigation was provided to avoid water stress. Weeds were manually removed where necessary, and fungicides and insecticides were applied once at anthesis stage to prevent diseases and insect damage. After harvest, the seeds were threshed, dried, the mature seeds were taken for quality trait determination.

### Wheat Quality Traits Measurement

In this study, a total of 9 wheat quality traits including six grain quality traits and three dough rheological properties were determined with a near-infrared analyzer DA7250 (Perten, Sweden), following the Chinese national standard (GB/T5498-1985), with the wheat quality standard curve constructed for NIR analyzer. Grain quality traits included Grain protein content (GPC, %), Grain test weight (GTW, g/L), Wet gluten content (WGC, %), SDS-sedimentation volume (SV, ml), Flour yield (FY, %) and Total starch content (TSC, %), and dough rheological properties included Dough development time (DDT, min), Dough stability time (DST, min) and Dough water absorption (DWA, %). All quality traits of 267 genotypes under the three environments of 2016–2017, 2017–2018 and 2018–2019 were measured.

To verify the accuracy of the wheat quality traits by the near-infrared analyzer, a Micro-doughLAB (Perten, Sweden) and a GM 2200 gluten analyzer (Perten, Sweden) were used to determine the three dough rheological properties (DDT, DST and DWA) and WGC of 50 representative wheat accessions in three years, which followed the American Association of Clinical Chemistry’ Standard AACC54-21 and AACC38-12, respectively (American Association of Cereal Chemists, 1995; American Association of Cereal Chemists, 2000). The methods used to verify SV and GTW were performed with reference to AACC56-61 and GB 5498-1985 (American Association of Cereal Chemists, 2000). FY was measured with a Buhler pneumatic laboratory mill following the GB/T 14614-2006. Considering that the NIR analyzer had been widely used in the determination of GPC and TSC, therefore, no verification test was designed for GPC and TSC.

### Statistical Analysis of the Phenotypic Traits

The best linear unbiased prediction (BLUP) values of all traits over three years were calculated by the R package Lme4 (Bates et al., 2014). The ANOVA of all quality traits in the three years (E1 to E3) and BLUP (E4) were analyzed using the software of SAS 8.0 (SAS Institute Inc., Cary, NC, USA), and the genotype and environment were treated as fixed and random, respectively. Statistical significance was determined when *P <0.05 and **P <0.01, respectively. The coefficient of variation (CV) was calculated by dividing the standard deviation by the average of traits. The generalized heritability (*h* ^2^) was calculated following the equation:

$$h2=\frac{Vg}{Vg+\frac{Vge}{l}+\frac{V\epsilon }{rl}}$$

where *Vg* is genotypic variance; *Vge* is the interaction variance between genotype and environment; *V*ϵ is the residual variance; *r* is the number of repeats in a single environment, and *l* is the number of environment. All values above were calculated using the R package Lme4 (Bates et al., 2014).

The correlation coefficients among all nine quality traits in four environments, and linear regression analysis between measured laboratory value and NIRS estimated value were calculated using the software of SPSS 19.0 (SPSS, Inc., Chicago, IL, USA).

### Genotyping and Genome-Wide Association Studies by Seven Models

Total genomic DNA was extracted from young leaves with a modified CTAB method (Priyadharshini et al., 2019), and the genotyping of 267 accessions was performed by 34,043 SNPs of the AxiomTM Wheat Breeder’s Genotyping Array (35K) by Capital Bio Technology Corporation, Beijing, China. After excluding the low-quality SNP markers with minor allele frequency (MAF) ≤0.05, missing data ≥20%, the proportion of heterozygous ≥20%, 19254 SNPs were used for GWAS. All SNP makers were anchored on the recent wheat genome (IWGSC RefSeq v1.1) using BLASTN by all SNP flanking sequences.

Six wheat grain quality traits and three dough rheological properties in four environments were simultaneously studied with two single-locus GWAS methods and five multi-locus GWAS methods. The single-locus GWAS was performed by Tassel 5.0 with two methods: GLM and MLM, and the multi-locus GWAS were performed by the R package mrMLM with five methods: mrMLM (Wang et al., 2016), FASTmrMLM (Tamba and Zhang, 2018), FastmrEMMA (Wen et al., 2017), pLARmEB (Zhang et al., 2017) and pKWmEB (Ren et al., 2018). Briefly, Population structure of 267 accessions generated by Admixture software was used as the Q matrix; the kinship (k) matrixes between the accessions used for single-locus GWAS and multi-locus GWAS were calculated by Tassel 5.0 and R package mrMLM, respectively; LOD >3.0 was used as the critical threshold for the significantly associated SNPs of multi-locus GWAS, and the standard Bonferroni correction (P = 0.05/19,254 = 2.56 × 10^−6^, or –log_10_P value = 5.59) was used as the threshold for single-locus GWAS.

### Phenotypic Difference Corresponding to QTNs and Prediction of Candidate Genes

All accessions were divided into two categories based on the genotype of each QTN for the significantly associated core QTNs detected by multiple methods or environments, and the effect of the genotype on the phenotype was verified using a t-test by SPSS 19.0. The boxplots were drawn using the R package ggplot2.

The latest wheat genome and gene annotation information (IWGSC RefSeq v1.1) were downloaded from Ensemble Plants database (http://plants.ensembl.org/info/website/ftp/index.html) and used for screening the candidate genes located in or near the candidate QTN regions that may regulate wheat quality traits. Simultaneously, transcripts per kilobase million (TPM) values were used to represent the expression levels of candidate genes. TPM values of candidate genes in the six tissues (root, leaf, peduncle, awn, glume and grain) were downloaded from the wheat expression database (http://www.wheat-expression.com) (Ramírez-González et al., 2018). Recently reported transcriptome data of embryo, endosperm and seed coat during wheat grain development were also used to analyze expression patterns of candidate genes to identify the possible regulatory network (Xiang et al., 2019).The original read count number matrix was obtained from the supplemental data of Xiang’s paper, and the TPM value was calculated using TBtools. Also, previous reports on QTLs and identified genes associated with quality traits of wheat and rice were used to select the candidate genes.

## Results

### Phenotypic Variations of Grain Quality Traits and Dough Rheological Properties

All the seven quality traits used for linear regression analysis showed good consistency between the NIRS prediction value and the laboratory measurement value, with R^2^ ranging from 0.50 of DWA in 2018 to 0.80 of WGC in 2017 (**Supplementary Table S2** and **Figure S1**). Among them, the correlation coefficients of grain quality traits were relatively high, with an average R^2^ from 0.65 (SV) to 0.89 (WGC), while that of the dough rheological properties were lower, from 0.52 (DWA) to 0.67 (DDT) (**Supplementary Table S2**). In general, it’s feasible to use NIRS analyzer to indirectly measure the quality traits.

**Figure 1:**
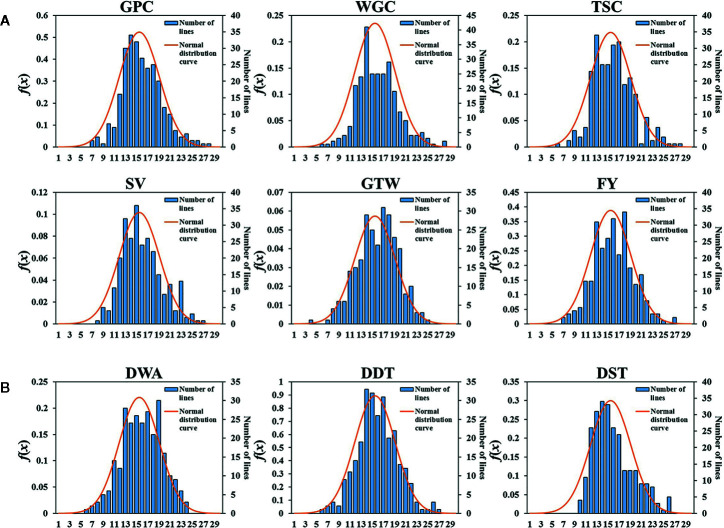
Histogram of the frequency distributions for the six grain quality traits and three dough rheological properties of wheat in BLUP. **(A)** Six grain quality traits, *GPC*, grain protein content; *WGC*, wet gluten content; *TSC*, total starch content; *SV*, SDS-sedimentation volume; *GTW*, grain test weight; *FY*, flour yield. **(B)** Three dough rheological properties, *DDT*, dough development time; *DST*, dough stability time; *DWA*, dough water absorption. The x-axis represents the number of class, the y-axis in left and right represent the normal distribution curve value and the number of sample, respectively.

Six grain quality traits and three dough rheological properties under four environments (2017, 2018, 2019, BLUP) showed approximately normal distributions (**Figure 1** and **Supplementary Figure S2**). Among them, the grain quality traits were more consistent, while the dough stabilization time (DST) showed a weak skewed distribution. The broad-sense heritability (*h* ^2^) of six grain quality traits in three years ranged from 68.21 to 76.14%, with the minimum of grain test weight (GTW) and the maximum of SDS-sedimentation volume (SV), indicating that the six grain quality traits were affected by environments to different degrees (**Table 1**). The coefficients of variation (CV) ranged from 0.88 to 20.22% for the six grain quality traits under four environments, with the minimum GTW in BLUP and the maximum of SV in 2019. Among the three dough rheological properties, in addition to the higher *h* ^2^ of dough development time (DDT) (77.27%), *h* ^2^ of the water absorption (WA) and DST were low, 67.92 and 50.35%, respectively (**Table 1**). Also, CV of DST in different environments ranged from 16.97 to 70.41%, indicating that the dough rheological properties were more susceptible to genotype × environment interactions. In general, there were significant differences among genotypes for all nine traits, indicating that it is suitable for multi-locus GWAS.

**Table 1:** Descriptive statistics of nine quality traits of wheat in three environments.

Traits^a^	Environment	Mean	SD^b^	Range	CV (%)^c^	*h^2^* (%)^d^	Genotype	Environment
GPC (%)	2016	13.70	1.29	10.65–19.69	9.40	71.85	**	**
	2017	14.99	1.45	11.23–19.84	9.70			
	2018	13.63	1.22	10.53–17.36	8.95			
	BLUP	14.11	0.76	12.12–16.57	5.39			
WGC (%)	2016	28.86	2.81	22.07–41.71	9.75	71.99	**	**
	2017	31.96	3.18	23.83–42.72	9.94			
	2018	28.58	2.82	20.41–37.73	9.87			
	BLUP	29.79	1.69	25.00–35.20	5.68			
TSC (%)	2016	56.15	3.03	47.57–69.23	5.40	73.87	**	**
	2017	59.93	3.25	51.29–71.42	5.42			
	2018	56.91	2.88	48.21–66.29	5.06			
	BLUP	57.66	1.83	52.48–63.67	3.17			
SV (ml)	2016	32.23	6.35	16.77–51.46	19.71	76.14	**	**
	2017	34.31	6.77	18.73–57.42	19.73			
	2018	27.75	5.61	14.87–43.44	20.22			
	BLUP	31.41	3.92	23.21–43.38	12.47			
GTW (g/L)	2016	783.57	14.49	738.50–824.00	1.85	68.21	**	**
	2017	792.78	14.11	739.00–821.50	1.78			
	2018	798.89	10.01	769.50–826.50	1.25			
	BLUP	791.74	6.94	769.29–808.51	0.88			
FY (%)	2016	69.55	1.59	63.00–73.50	2.28	71.44	**	**
	2017	67.30	2.19	62.00–80.25	3.25			
	2018	71.19	1.57	66.00–76.00	2.20			
	BLUP	69.35	1.03	66.72–72.55	1.48			
DWA (%)	2016	61.83	4.05	53.85–71.80	6.54%	67.92%	**	**
	2017	62.54	3.32	54.15–70.80	5.31%			
	2018	61.62	2.74	53.80–69.45	4.45%			
	BLUP	61.99	1.81	56.86–65.96	2.91%			
DDT (%)	2016	2.87	0.76	0.75–5.45	26.36%	77.27%	**	**
	2017	3.82	0.61	1.70–5.80	16.01%			
	2018	2.93	0.71	1.05–4.90	24.26%			
	BLUP	3.20	0.45	1.98–4.51	13.91%			
DST (%)	2016	11.26	4.32	1.30–21.80	38.32%	50.35%	**	**
	2017	9.01	4.69	0.45–24.20	52.10%			
	2018	4.61	3.25	0.15–14.85	70.41%			
	BLUP	7.82	1.33	5.54–11.64	16.97%			

^a^GPC, grain protein content; WGC, wet gluten content; TSC, total starch content; SV, SDS-sedimentation volume; GTW, grain test weight; FY, flour yield; DWA, dough water absorption; DDT, dough development time; DST, dough stability time. ^b^Standard Deviation. ^c^Coefficient of Variation. ^d^Broad-sense Heritability. **Significance at p <0.01.

To explain the relationships between different quality traits, both Spearman and Pearson approaches were used to examine the basic correlations of the nine quality traits in the four environments (**Table 2** and **Supplementary Table S3**). GPC, WGC, TSC and SV of grain quality traits all showed significant and positive correlations each other by the two methods (0.70–0.98), and the correlation coefficients among GPC, WGC and TSC were above 0.9. However, GPC, WGC, TSC and SV were negatively correlated with GTW and FY (−0.13 to −0.43), and GTW and FY were significantly and positively correlated (0.28–0.31). All three dough rheological properties were significantly and positively correlated (0.314–0.504), and both DDT and DST were significantly and positively correlated with GPC, WGC, TSC and SV. The difference was that DDT was positively correlated with GTW, while DST was significantly and negatively correlated with GTW. DWA was significantly correlated with the other five grain quality traits except for GPC. The significant correlation between nine quality traits implied that they might be regulated by multiple co-effect loci.

**Table 2:** Correlations between six grain quality traits and three dough rheological properties of wheat across three environments and BLUP values.

		Grain quality traits						Dough rheological properties		
		GPC	WGC	TSC	SV	GTW	FY	DWA	DDT	DST
Grain quality traits	GPC	1	0.97**	0.92**	0.70**	−0.13*	−0.40**	−0.03	0.68**	0.35**
	WGC	0.98**	1	0.97**	0.74**	−0.17**	−0.33**	0.15*	0.73**	0.37**
	TSC	0.94**	0.98**	1	0.84**	−0.20**	−0.24**	0.26**	0.81**	0.47**
	SV	0.73**	0.77**	0.85**	1	−0.25**	−0.13*	0.43**	0.80**	0.70**
	GTW	−0.15*	−0.21**	−0.24**	−0.27**	1	0.28**	−0.21**	0.20**	−0.28**
	FY	−0.43**	−0.37**	−0.29**	−0.17**	0.31**	1	0.35**	−0.01	−0.07
Dough rheological properties	DWA	−0.02	0.15*	0.24**	0.42**	−0.24**	0.36**	1	0.32**	0.31**
	DDT	0.71**	0.75**	0.82**	0.81**	0.20**	−0.02	0.31**	1	0.50**
	DST	0.33**	0.35**	0.44**	0.67**	−0.29**	−0.06	0.33**	0.47**	1

GPC, grain protein content; WGC, wet gluten content; TSC, total starch content; SV, SDS-sedimentation volume; GTW, grain test weight; FY, flour yield; DWA, dough water absorption; DDT, dough development time; DST, dough stability time.

Spearman and Pearson correlation coefficients and were listed above and below the diagonal, respectively.

* and **Significance at p <0.05, 0.01, respectively.

### QTNs for Grain Quality Traits and Dough Rheological Properties

A total of 19,254 high-quality SNPs were screened from 34,043 SNPs as the genotype data with stringent parameters (MAF ≥0.05, missing data ≤20%, the proportion of heterozygous ≤20%); the optimal number of sub-populations (*k*) was determined as 3; the phenotypic values in four environments (2017, 2018, 2019, BLUP) were used as phenotype data. Earlier studies showed that QTNs within 5 Mb or less were considered to be caused by a single gene (Visscher et al., 1996; Swanson-Wagner et al., 2009; Wang et al., 2012). Considering the longer linkage disequilibrium attenuation distance of wheat, QTNs within 7 Mb was viewed as a QTN region (Appels et al., 2018). As a result, 299 QTNs within 105 genomic regions were significantly associated with six grain quality traits and three dough rheological properties at the critical LOD ≥3 (**Supplementary Table S4**). QTN regions were named based on their physical location on the chromosome, for example, q1A-1 represented the first QTN region on chromosome 1A, and q5B-4 represented the fourth QTN region on chromosome 5B. The number of QTNs detected in four environments were 114, 84, 65 and 125, respectively; the number of significant QTNs varied across various traits and the environments, and more significant QNTs were identified for grain quality traits (**Supplementary Table S4**). A total of 246 QTNs associated with grain quality traits were identified, from 39 for FY to 96 for GPC, while only 86 QTNs were significantly associated with the three dough rheological properties, from nine for DST to 42 for DDT (**Table 3**). There were 33 QTNs associated with both grain quality traits and dough rheological properties, and more than half of them (22 of 33) were related to DDT, which was consistent with the high correlation coefficient between DDT and grain quality traits.

**Table 3:** The number of significant QTNs for six grain quality traits and three dough rheological properties of wheat by two single-locus and five multi-locus GWAS methods.

Type of traits	Traits^a^	Multi-locus GWAS					Single-locus GWAS		Total
		mrMLM	FASTmrMLM	FASTmrEMMA	pLARmEB	pKWmEB	GLM	MLM	
Grain quality traits	GPC	11	16	10	16	24	54	0	96
	TSC	9	25	6	19	22	46	0	88
	WGC	14	17	11	19	24	42	0	83
	GTW	12	13	5	18	19	7	0	50
	SV	12	19	6	14	23	10	0	61
	FY	10	13	5	14	15	9	1	39
	Total	71	73	30	70	91	41	0	246
Dough rheological properties	DWA	11	18	10	17	16	3	1	37
	DDT	6	19	7	18	22	3	0	42
	DST	2	2	5	3	4	0	0	9
	Total	21	39	22	38	41	6	1	86
Total		89	104	46	98	118	81	2	299

^a^GPC, grain protein content; WGC, wet gluten content; TSC, total starch content; SV, SDS-sedimentation volume; GTW, grain test weight; FY, flour yield; DWA, dough water absorption; DDT, dough development time; DST, dough stability time.

**Figure 2:**
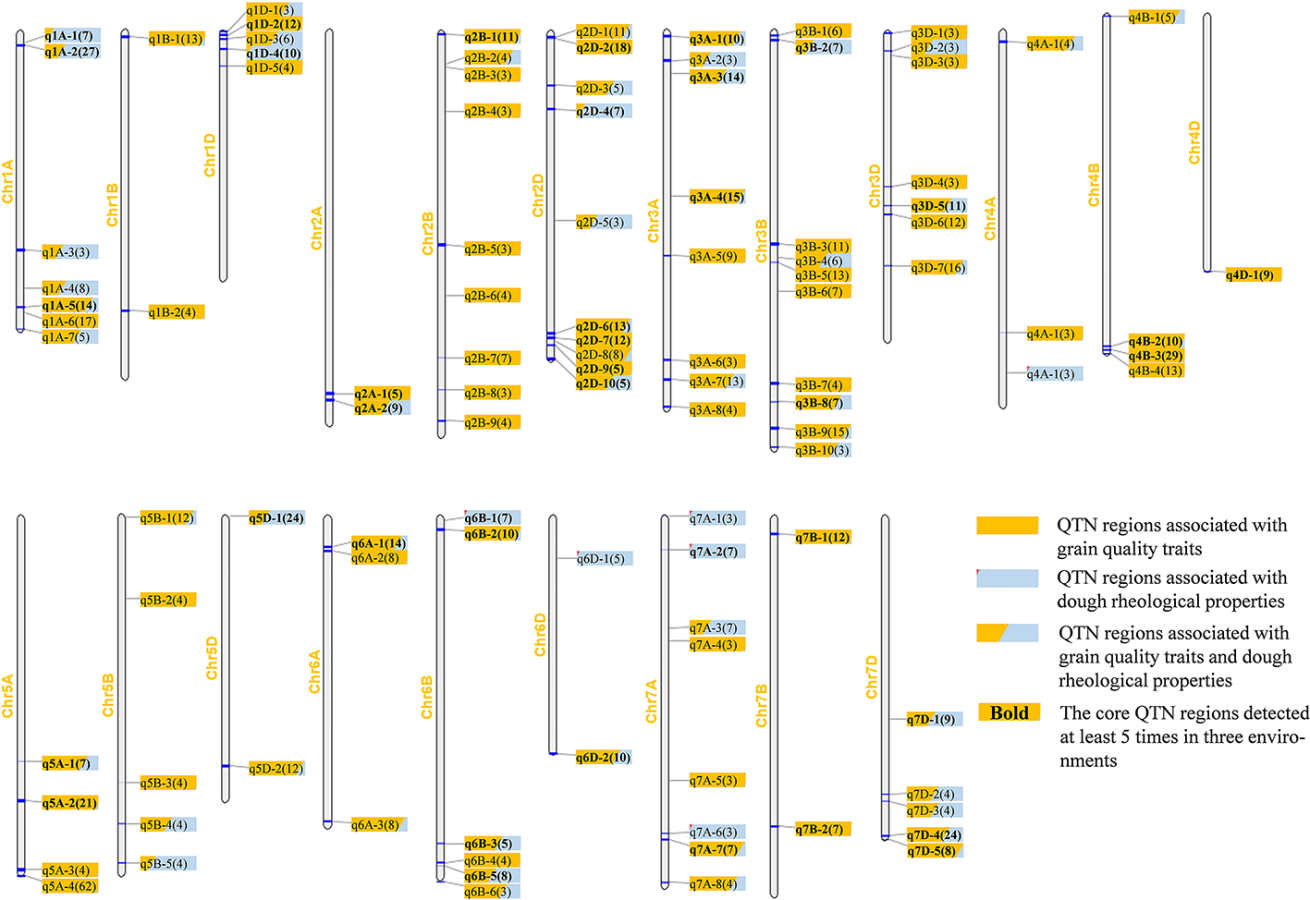
Chromosomal distribution of 105 QTN regions significantly associated with nine wheat quality traits. Different shades of color are used to represent QTN regions associated with different categories of quality traits, light blue for dough rheological properties, orange for grain quality traits, and combinations of light blue and orange for both types of traits. The shaded area represents the number of QTNs associated with the two types of traits in each QTN region. The number in parentheses represents the total number of times this region has been detected, and the bold font represents the 40 core QTN regions.

There were 105 QTN regions identified at least three times in two environments (**Supplementary Table S5**). These regions were unevenly distributed on 21 wheat chromosomes, at least one on chromosome 4D, and at most 10 on chromosomes 2B and 2D, which indicated that the chromosomes 2B and 2D may have a more contribution to wheat quality (**Figure 2**). The number of QTNs in the QTN region ranged from 1 to 21, which was related to the uneven distribution of high-quality SNP makers across the wheat genome (**Supplementary Tables S4** and **S5**).

Of the 105 QTN regions, more than half of them (55%, 58/105) were detected more than five times, of which 28 QTN regions were found more than 10 times (**Supplementary Table S4**). Forty regions were significantly correlated with six grain quality traits, six regions with dough rheological properties, and the other 59 regions were correlated with these two types of traits (**Figure 2**).

**Figure 3:**
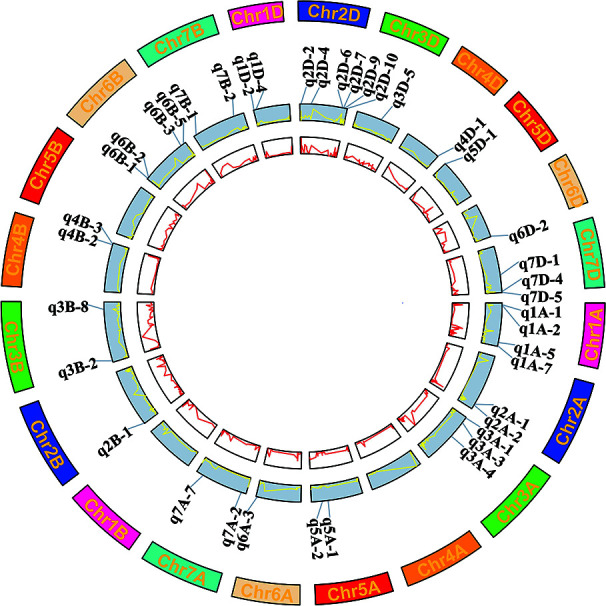
Wheat chromosomes with the 40 core QTN regions significantly associated with nine wheat quality traits. The inside two circles with red and yellow lines represents the LOD and R^2^ value curves cross three environments and BLUP values.

There were 40 QTN regions detected more than five times in three environments, which were considered as stable core QTN candidate regions (**Figure 3** and **Table 4**). These core QTN regions could explain an average of 9.66% of phenotypic variation, and the R^2^ ranged from 4.38 to 18.69%, with a maximum of q2A-1 explaining a phenotypic variation of DST of 18.69%, indicating that this region may be a relatively major QTL. The LOD values ranged from 3.16 to 12.9, with an average of 6.45. q5D-1 had the maximum LOD value and could still explain the phenotypic variation of DWA of 17.96% (R^2^) (**Table 4**). There were 14 QTN regions significantly associated with at least two traits in two environments, of which seven regions were associated with grain quality traits, and the remaining seven regions were associated with both grain quality traits and dough rheological properties. These regions account for the phenotypic variation of 5.63 to 16.75% and 6.71 to 18.69%, respectively (**Table 4**). It is worth noting that five QTN regions (q1A-2, q2D-7, q3A-4, q5A-2 and q7D-4) were significantly associated with three to five traits in two environments.

**Table 4:** Details of 40 core QTN regions associated with 9 quality traits *via* single-locus and multi-locus GWAS in wheat.

Region^a^	Chr.^b^	SNP^c^	Pos. (bp)^d^	No.^e^	*r* ^2^ (%)^f^	*LOD*	Positions (Mb)	Method^g^	Trait–environment^h^
q1A-1	1A	AX-95630408	24247379	1	11.17	9.39	24.25	2, 4, 5	TSC_E1, DDT_E1, DDT_E3, DDT_E4
q1A-2	1A	AX-94694208	32088842	5	18.69	9.92	32.09	1, 2, 3, 4, 5	TSC_E1, TSC_E3, SV_E1, SV_E3, SV_E4, WGC_E3, DDT_E3, DDT_E4, DST_E2, DST_E3, DST_E4
q1A-5	1A	AX-94385896	540656564	3	6.18	8.39	540.66–544.61	1, 2, 4, 5	GPC_E1, TSC_E1, WGC_E1, SV_E2, DDT_E4
q1A-7	1A	AX-94592638	584683041	2	5.76	3.67	584.68–586.51	2, 5	DDT_E2, GPC_E1, TSC_E1, TSC_E4
q1D-2	1D	AX-94685030	7347635	6	5.63	4.78	6.77–11.62	5, 6	SV_E1, SV_E2, SV_E4, TSC_E4, WGC_E4, GTW_E2, GTW_E4, GPC_E4, FY_E4
q1D-4	1D	AX-94926263	34945066	2	5.35	7.84	34.95–38.66	1, 2, 3, 4, 5	DWA_E1, DWA_E4, SV_E3
q2A-1	2A	AX-94564840	710194426	3	17.38	7.77	710.19–716.49	1, 2, 5	SV_E1, FY_E3, FY_E4
q2A-2	2A	AX-94732478	728028914	3	9.85	11.22	723.10–729.20	2, 5, 6	TSC_E1, WGC_E1, WGC_E2, DDT_E1, DDT_E4, SV_E1, GPC_E2
q2B-1	2B	AX-95024633	6210320	3	6.00	6.77	5.67–9.89	1, 2, 5	WGC_E2, WGC_E4, GPC_E2, SV_E3, GPC_E4, DWA_E2, TSC_E4
q2D-2	2D	AX-94882684	16344549	5	8.07	6.91	16.34–17.63	1, 2, 3, 4, 5, 6, 7	WGC_E3, WGC_E4, SV_E3, GPC_E3, TSC_E3, FY_E2
q2D-4	2D	AX-94752964	152008074	2	7.17	3.27	152.01–156.83	2, 3, 4, 5	DWA_E1, DWA_E3, DWA_E4, FY_E2
q2D-6	2D	AX-95217872	591022287	6	16.75	3.16	591.02–596.91	1, 2, 3, 4, 5, 6	GPC_E1, GPC_E4, WGC_E1, WGC_E4, FY_E1, DDT_E1, SV_E3, TSC_E3, DST_E3
q2D-7	2D	AX-94945383	605327679	3	11.62	6.86	600.03–605.33	1, 2, 4, 5	GPC_E2, GPC_E3, GPC_E4, WGC_E2, WGC_E4, TSC_E2, TSC_E3, GTW_E2
q2D-9	2D	AX-94429731	615471428	2	10.55	4.15	615.47–618.90	1, 5	SV_E1, SV_E3, TSC_E2, WGC_E2, GPC_E2
q2D-10	2D	AX-95169570	646594590	5	8.40	5.37	641.11–647.28	1, 2, 5	SV_E4, FY_E1, DDT_E2, DST_E4
q3A-1	3A	AX-94739609	15691226	5	11.68	5.77	10.30–15.69	1, 2, 3, 5	SV_E4, SV_E3, GPC_E1, WGC_E1, DDT_E1, GTW_E3
q3A-3	3A	AX-95075882	86369509	1	15.50	7.13	86.37	1, 2, 3, 4, 5, 6	GTW_E1, GTW_E2, GTW_E4, DWA_E2, DWA_E4
q3A-4	3A	AX-94710748	326464823	1	8.82	9.13	326.46	2, 4, 5	GPC_E2, GPC_E4, TSC_E2, WGC_E2, WGC_E4, DDT_E2, DDT_E3
q3B-2	3B	AX-94656528	18247590	4	7.31	7.61	18.25–23.60	2, 3, 4, 5	DWA_E1, DWA_E3, DWA_E4, GTW_E4
q3B-8	3B	AX-94562553	728919189	5	12.15	3.82	728.92–730.76	1, 6	TSC_E4, WGC_E4, GTW_E4, DWA_E2, DST_E1
q3D-5	3D	AX-94382102	342521649	2	6.71	6.03	342.52–344.34	2, 3, 4, 5, 6	SV_E2, SV_E4, GTW_E2, DWA_E2, DWA_E4, DDT_E3, DST_E3
q4B-2	4B	AX-95120436	651799357	6	7.57	10.37	650.27–653.40	1, 2, 4, 5	FY_E1, FY_E4, SV_E1, DDT_E3, GTW_E2, GTW_E4
q4B-3	4B	AX-94694411	660719506	3	9.51	7.26	657.47–660.72	1, 2, 3, 4, 5, 6	GPC_E1, GPC_E4, TSC_E4, WGC_E4, SV_E4, DST_E3
q4D-1	4D	AX-95202302	665814724	2	6.81	5.55	504.51–506.72	2, 3, 4, 5, 6	GTW_E2, GTW_E4, GPC_E1, TSC_E1, WGC_E1
q5A-1	5A	AX-94452354	481670094	3	6.38	3.87	481.67–482.35	3, 4, 5	GTW_E1, GTW_E2, GTW_E4, DWA_E1, DDT_E1, FY_E2
q5A-2	5A	AX-94399903	561173979	3	9.57	7.23	556.01–562.48	2, 3, 4, 5, 6	GPC_E2, GPC_E3, WGC_E2, WGC_E3, TSC_E2, TSC_E3, TSC_E4, SV_E2, DST_E4
q5D-1	5D	AX-94991433	3609894	1	17.96	12.90	3.61	1, 2, 3, 4, 5, 6, 7	DWA_E1, DWA_E2, DWA_E3, DWA_E4, FY_E2, FY_E4
q6A-3	6A	AX-94643554	602915841	2	12.25	6.11	599.24–602.92	1, 4, 5	GTW_E2, GTW_E4, DWA_E2, GPC_E3, TSC_E3, WGC_E3
q6B-1	6B	AX-94439435	11298582	1	6.99	9.23	11.3	2, 3, 4, 5	DWA_E1, DWA_E4
q6B-2	6B	AX-94587213	27999566	3	17.73	3.62	26.59–32.20	1, 2, 4, 5	GTW_E1, GTW_E3, GTW_E4, FY_E3, GPC_E3, DST_E3
q6B-3	6B	AX-94497531	643251027	2	5.81	5.03	643.25–645.53	2, 5	DWA_E1, DWA_E4, TSC_E3, WGC_E3
q6B-5	6B	AX-94661897	688297021	2	6.68	4.73	687.55–688.30	1, 2, 4, 5	DWA_E3, DWA_E4, FY_E1, GPC_E1, TSC_E1
q6D-2	6D	AX-94472993	467833307	7	10.33	5.39	465.96–470.63	1, 2, 4, 5	DDT_E1, DDT_E2, DWA_E4, GTW_E2, FY_E1, TSC_E1, GPC_E2, WGC_E2
q7A-2	7A	AX-94601136	69901493	2	5.08	3.95	69.36–69.90	2, 4, 5	DWA_E1, DWA_E3, DWA_E4
q7A-7	7A	AX-94821471	636230293	4	4.38	3.49	634.91–638.71	2, 4, 5	GTW_E1, GTW_E3, GTW_E4, SV_E1, DDT_E1
q7B-1	7B	AX-95203056	38626693	2	9.41	7.06	33.90–38.63	1, 2, 3, 4, 5	TSC_E2, TSC_E4, WGC_E2, WGC_E4, GPC_E2, FY_E3
q7B-2	7B	AX-94497402	607330964	2	8.74	6.32	607.33–611.72	2, 3, 4, 5, 6	FY_E1, FY_E2, FY_E3, FY_E4
q7D-1	7D	AX-94482935	398719475	1	7.88	7.41	398.72	1, 2, 3, 4, 5	DWA_E1, SV_E2, SV_E4
q7D-4	7D	AX-94956586	626051127	4	15.91	5.54	626.05–629.87	1, 2, 3, 4, 5, 6	TSC_E1, TSC_E4, SV_E1, SV_E3, SV_E4, GPC_E1, GPC_E4, WGC_E1, WGC_E4, DDT_E1, DDT_E4
q7D-5	7D	AX-94613317	634138826	3	6.53	3.83	634.14–635.56	1, 2, 3, 4, 5, 6	FY_E2, FY_E3, WGC_E1, TSC_E1, DDT_E1, GTW_E2

^a^Core QTN regions which was detected at least in three environments five times. ^b^Chromosome. ^c^QTNs that were most significantly associated with the trait. ^d^QTN position (bp) on wheat genome assembly IWGSC refseq v1.1. ^e^The number of significant QTNs identified in the region. ^f^The proportion of phenotypic variance explained by the most significant QTN in each region. ^g^The mrMLM, FASTmrMLM, FASTmrEMMA, pLARmEB, pKWmEB, GLM and MLM methods were marked from 1 to 7, respectively. ^h^The trait–environment combination of QTN. Traits are defined in **Tables 1** and **2**, and E1 to E4 denote 2016, 2017, 2018 and BLUP, respectively.

Fifteen QTN regions showed a stable association with the same traits in at least three environments. Of the 15 QTN regions, nine regions (q1D-2, SV; q2D-7, GPC; q3A-3, GTW; q5A-1, GTW; q5A-2, TSC; q6B-2, GTW; q7A-7, GTW; q7B-2,FY; q7D-4, SV) were related to grain quality traits, and five regions (q1A-1, DDT; q2D-4, DWA; q3B-2, DWA; q5D-1, DWA; q7A-2, DWA) were related to dough rheological properties, which explained the phenotypic variation of 4.38 to 17.72% and 5.08 to 17.96%, respectively (**Table 4**). Notably, q5D-1, q7B-2 showed significant correlation with an R^2^ of 17.96 and 8.74% for DWA and FY respectively in all four environments. Furthermore, there were certain QTN regions, such as q2D-1, with LODs of 10.07 and R^2^ values of 14.65 and q6A-1, with LOD values of 11.73, and R^2^ value of 13.36, with high LODs or R^2^ values outside the 40 core QTN regions (**Figure 3** and **Supplementary Table S5**). Although these QTN regions were excluded due to their low stability in multiple environments, they could still be used as a secondary candidate locus for subsequent analysis.

### Phenotypic Difference Corresponding to QTNs

**Figure 4:**
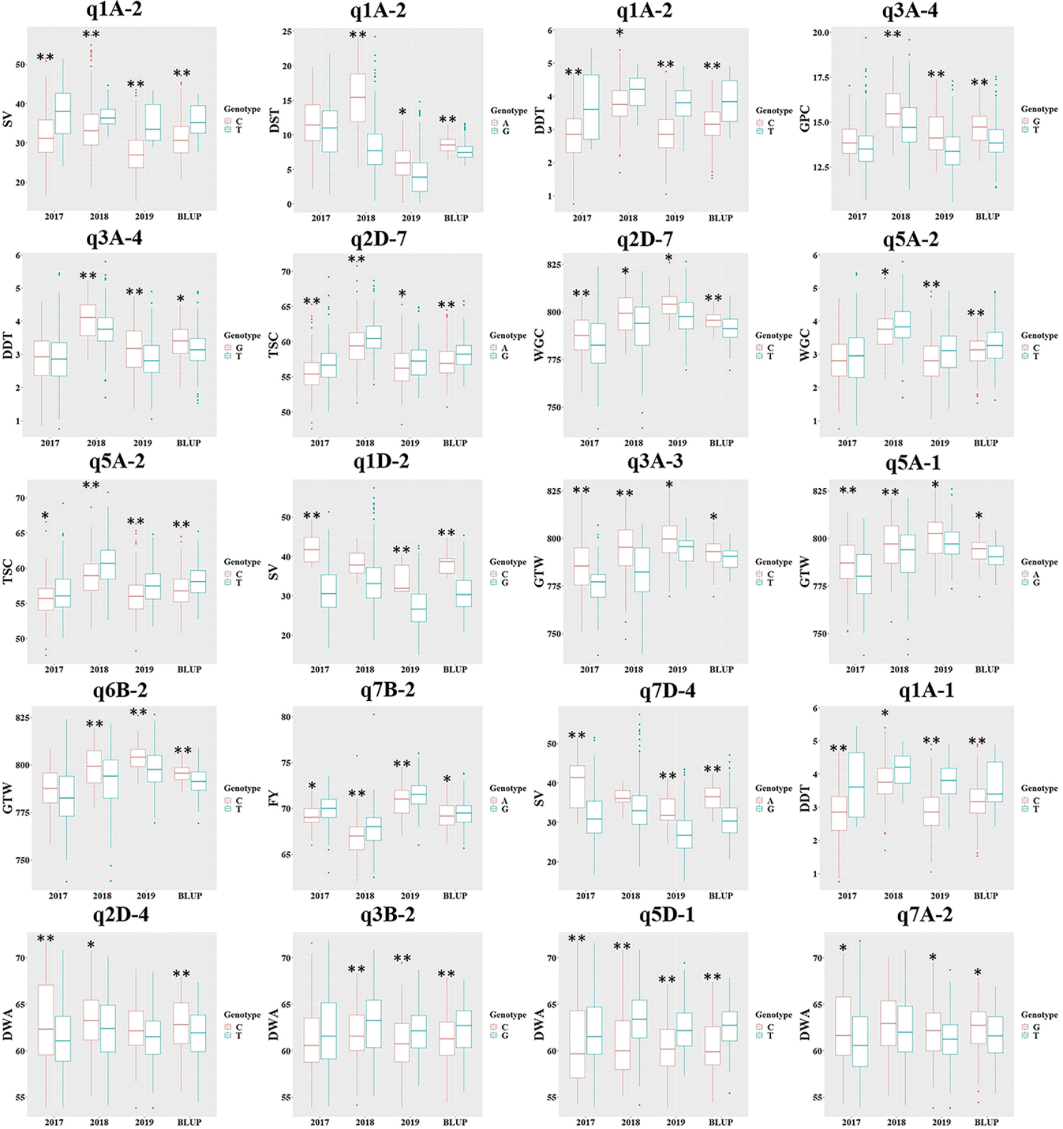
The phenotypic difference of the related traits between the two kinds of genotypes of 15 core QTN regions. *GPC*, grain protein content; *WGC*, wet gluten content; *TSC*, total starch content; *SV*, SDS-sedimentation volume; *GTW*, grain test weight; *FY*, flour yield; *DDT*, dough development time; *DST*, dough stability time; *DWA*, dough water absorption. The SNPs used for these loci were AX-94412818 (q1A-2), AX-94694208 (q1A-2), AX-94412818 (q1A-2), AX-94710748 (q3A-4), AX-94710748 (q3A-4), AX-94945383 (q2D-7), AX-94945383 (q2D-7), AX-94399903 (q5A-2), AX-94399903 (q5A-2), AX-94966191 (q1D-2), AX-95075882 (q3A-3), AX-94530985 (q5A-1), AX-94879817 (q6B-2), AX-94497402 (q7B-2), AX-94613939 (q7D-4), AX-95630408 (q1A-1), AX-94860457 (q2D-4), AX-94791594 (q3B-2), AX-94530985 (q5D-1) and AX-94601136 (q7A-2), where the QTN region was in the parentheses. * and **Significance at P <0.05, 0.01, respectively.

To test the effect of different genotypes on traits, 16 reliable QTNs in 15 core QTN regions were selected to group the populations according to their genotypes, and a t-test was used to test the significance of genotype effect on the traits (**Figure 4**). All 16 QTNs in 15 core regions revealed significant differences (at P <0.05) of the traits between the two genotypes in at least three environments, and the eight QTNs in the eight core regions had significant differences in all four environments (q1A-2, AX-94412818; q2D-7, AX-94945383; q5A-2, AX-94399903; q3A-3, AX-95075882; q5A-1, AX-94530985; q7B-2, AX-94497402; q1A-1, AX-95630408; q3B-2, AX-94791594), which indicated that these QTNs had a great influence on phenotypic variation (**Figure 4**). Among the 15 core regions, q1A-2 and q3A-4 had effects on both grain quality traits and dough rheological properties (q1A-2, SV&DST&DDT; q3A-4, GPC&DDT), and q2D-7 and q5A-2 had effects on WGC and GPC. These four regions may contain key candidate genes that could regulate wheat quality as a whole. Furthermore, loci AX-94412818 (q1A-2) and AX-94791594 (q3B-2) had significant effects at P <0.01 on traits in all environments (**Figure 4**), and could be used as optimal loci in marker-assisted breeding and quality improvement.

### Preliminary Validation of QTN Regions and Prediction of Candidate Genes

Based on the integrated genetic map and the physical position of the QTL on the chromosomes, we investigated whether the significantly associated QTN regions identified in this study were the same or close to the previously identified QTLs (**Supplementary Table S6**). It was observed that 21 of the 40 core QTN regions were consistent with previous reports. For instances, q1A-1, q1A-2, q1D-4, q2A-2, q2D-4, q2D-10, q5D-1 and q7D-4 were consistent with QTLs associated with dough rheological properties reported previously, while q1A-7, q1D-2, q2A-1, q2B-1, q2D-6, q2D-7, q3A-4, q3B-2, q4B-2, q6B-2, q6D-2 and q7A-7 were consistent with QTLs associated with grain quality traits (Kuchel et al., 2006; Mccartney et al., 2006; Li et al., 2013; Tsilo et al., 2013). These QTN regions consistent with previously reported QTLs explained the phenotypic variation of 4.38 to 18.69%, with an average of 10.63%. The remaining 19 QTN regions were considered as new QTLs controlling wheat quality traits, which could explain the phenotypic variation of 5.08 to 15.5%, with an average of 8.2% (**Table 4**).

**Figure 5:**
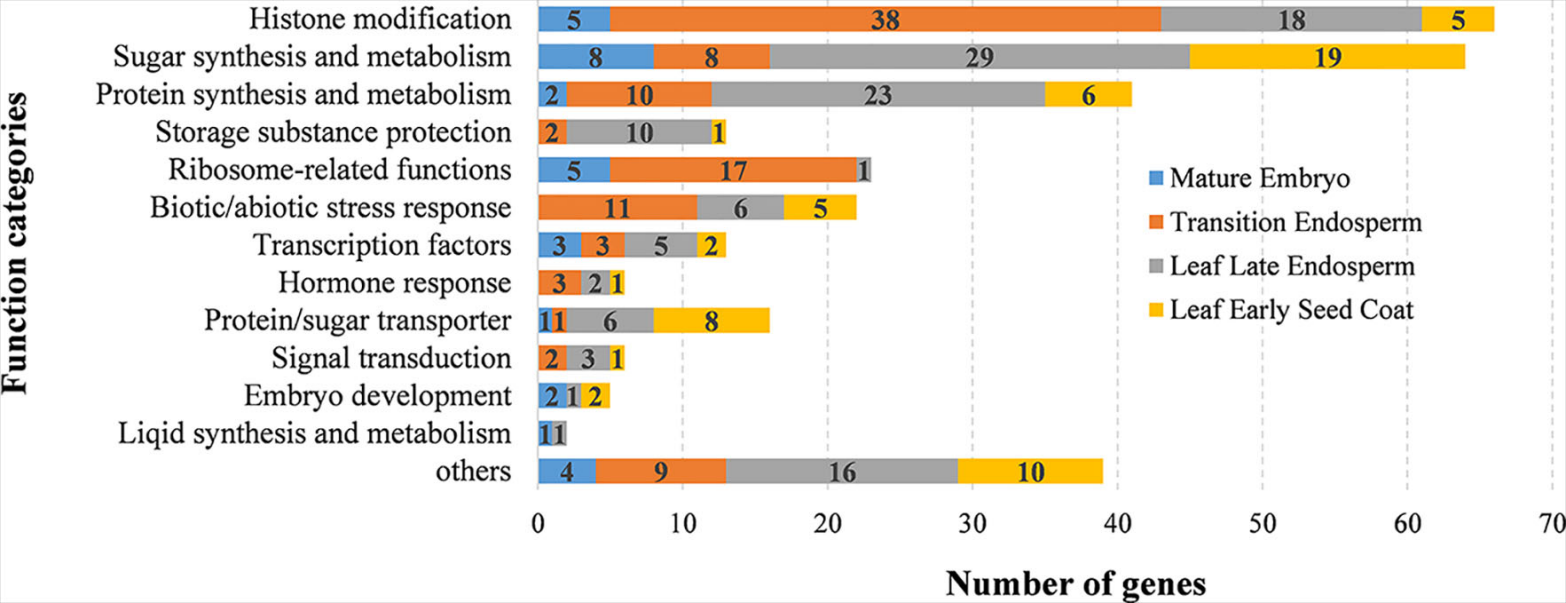
The functional distribution of the 318 candidate genes in the QTN regions identified. The left side represents the function categories based on function annotations by BLASTP to NR database. The different colors in the columns represent the number of genes specifically expressed in different tissues during wheat grain development.

**Figure 6:**
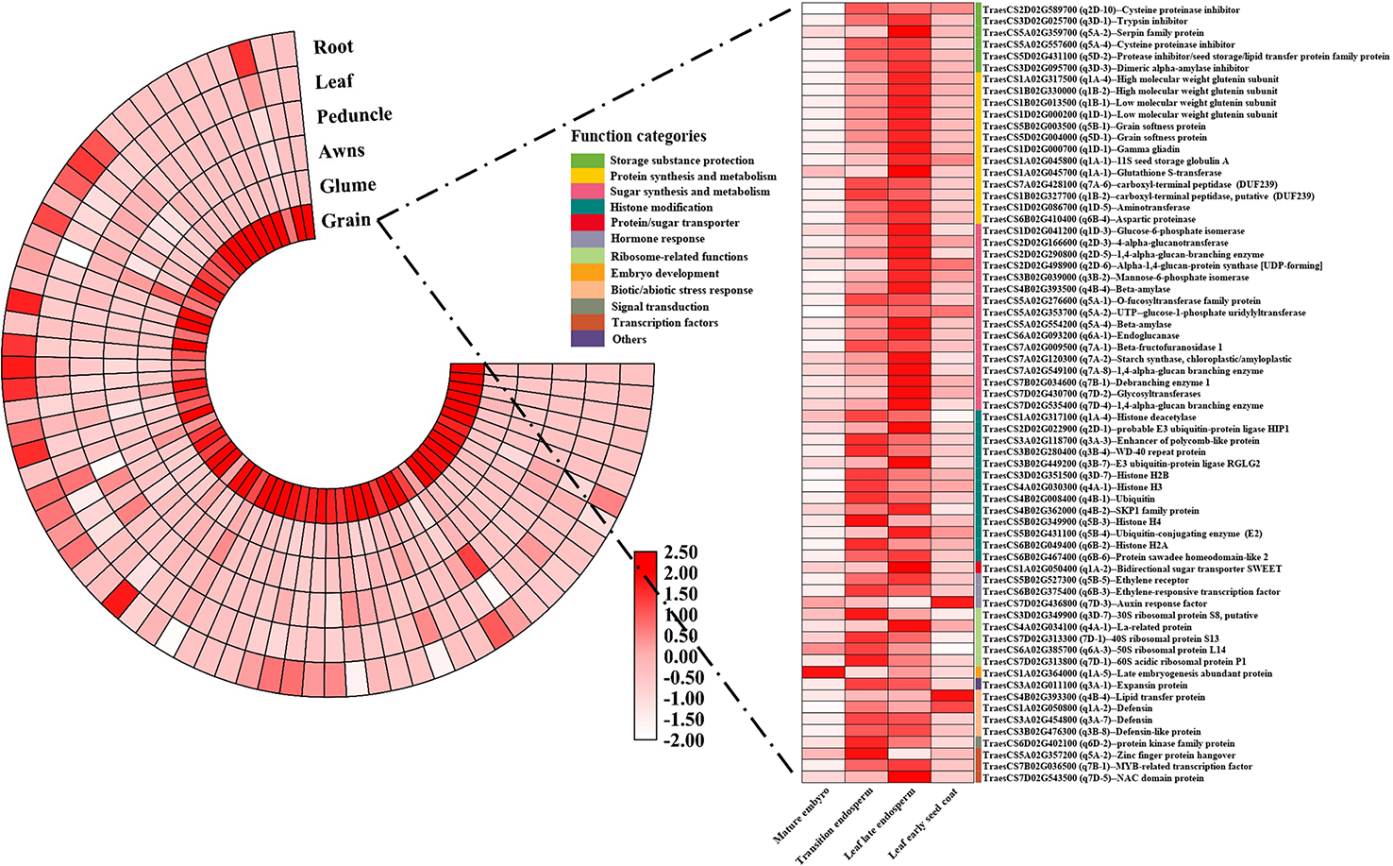
The tissue specific expression patterns of the 60 core candidate genes affecting wheat quality traits. The depth of color reflected the log_2_(TPM+1) value and gene annotation information were obtained from Nr database using a BLASTN program.

To further analyze the candidate genes in each QTN region, based on the wheat gene structure and function annotation information, after the manual screening, combined with the expression information in six tissues, a total of 318 candidate genes stably expressed in grain were found in or near the 101 QTN regions (**Supplementary Table S7**). Based on the combination of gene function annotation and existing knowledge of quality formation pathways, the functions that these candidate genes may participate in were temporarily classified (**Figure 5**). Overall, genes involved in protein synthesis and metabolism, sugar synthesis and metabolism, protein/sugar transporter, histone modification, ribosome-related and transcription factor accounted for a large proportion, which may significantly affect the development of grain and the formation of quality traits (**Figure 5**). To further speculate the possible roles of these candidate genes in the formation of wheat grain quality, a published RNA-seq dataset of wheat grain development was used to identify the expression characteristics of these genes in different parts of the grain (**Supplementary Table S7**) (Xiang et al., 2019). More than 70% (223/318) of candidate genes were preferentially expressed in endosperm, and they were mainly concentrated in sugar/protein synthesis, storage substance protection, transcription factors, histone modifications, biotic/abiotic stress response and ribosomal-related functional categories (**Figure 5**). Among them, a large number of histone modification, ribosomal-related and biotic/abiotic stress response functional genes were mainly high expressed at the early stage of endosperm development (transition endosperm), while sugar/protein synthesis, storage substance protection and transcription factor genes were highly expressed at the late stage of endosperm development (leaf late endosperm). Most of ribosome-related genes (74%, 17/23) were highly expressed at early endosperm development to construct a basic framework for storage protein synthesis (**Figure 5**). While, 56% (23/41) of protein synthesis-related genes, 45% (29/64) of sugar synthesis-related genes, and 77% (10/13) of storage substance protection-related genes were specifically overexpressed in leaf late endosperm, indicating these genes played indispensable roles at the late stage of endosperm development in the formation of grain quality (**Figures 5** and **6**). Genes involved in histone modification were preferentially expressed at the early stage of endosperm development, In contrast, more transcription factor genes were highly expressed at the late stage, which indicated that the regulation of transcription factors on genes for storage substance synthesis or protection might be more direct.

Based on gene function categories and expression levels during grain development, 67 representative core candidate genes were found, and their function annotations and expression information were shown in **Figure 6**. All 67 genes were preferentially expressed in the grain, except for four genes which were highly expressed in embryo or seed coat, the other 63 genes were highly expressed in the endosperm. Thirty-five genes with specific high expression in the leaf late endosperm, which were involved in the synthesis of storage sugar/protein and the storage substance protection, were selected and believed to have a direct contribution to the formation of wheat grain quality, such as high/low molecular weight glutenin subunits (*TraesCS1B02G330000*, *TraesCS1D02G000200*), grain softness protein (*TraesCS5D02G004000*), aspartic proteinase (*TraesCS6B02G410400*), Glucose-6-phosphate isomerase (*TraesCS1D02G041200*), beta-amylase (*TraesCS5A02G554200*), and protease inhibitor/seed storage/lipid transfer protein family protein (*TraesCS5D02G431100*). Several genes involved in histone modification were also determined, such as histone deacetylase (*TraesCS1A02G317100*), probable E3 ubiquitin-protein ligase HIP1 (*TraesCS2D02G022900*), Ubiquitin-conjugating enzyme (E2) (*TraesCS5B02G431100*) and Protein sawadee homeodomain-like 2 (*TraesCS6B02G467400*). Additionally, many genes related to hormone response, abiotic/biotic stress response, transcription factors and sugar transport were preserved, such as bidirectional sugar transporter SWEET (*TraesCS1A02G050400*), ethylene receptor (*TraesCS5B02G527300*), ethylene-responsive transcription factor (*TraesCS6B02G375400*), defensin-like protein (*TraesCS3B02G476300*) and MYB-related transcription factor (*TraesCS7B02G036500*). Furthermore, among the 67 core candidate genes, the homologous genes of eight candidate genes in rice have been proved to be related to rice quality traits (**Table 5**). The functions of these eight genes in rice and wheat may be conservative, and they can be used for wheat quality improvement preferentially.

**Table 5:** Details of wheat candidate genes in QTN regions that have been reported in rice.

Candidate gene ID In wheat^a^	Region associated	Chr	Gene Position	Homologuein Rice^b^	Homologue ID in Rice^c^	Reference	Annotation by Nr database
TraesCS1D02G041200	q1D-3	1D	20.04	*OsG6PIb*	AK068236	Peng et al., 2014	Glucose-6-phosphate isomerase
TraesCS1D02G086700	q1D-5	1D	71.28	*OsAlaAT*	AK102488	Peng et al., 2014	Aminotransferase
TraesCS2D02G290800	q2D-5	2D	372.92	*OsSBEIIa*	AB023498	Peng et al., 2014	1,4-alpha-glucan-branching enzyme
TraesCS5D02G431100	q5D-2	5D	488.20	*OsLTPL36*	Os03g0369100	Wang et al., 2015	Protease inhibitor/seed storage/lipid transfer protein family protein
TraesCS7A02G549100	q7A-8	7A	723.26	*OsSBE1*	AK119436	Peng et al., 2014	1,4-alpha-glucan branching enzyme
TraesCS7B02G034600	q7B-1	7B	33.91	*OsPullulanase*	AB012915	Peng et al., 2014	Debranching enzyme 1
TraesCS7D02G436800	q7D-3	7D	556.12	*OsARF10*	Os06g0685700	Huang et al., 2016	Auxin response factor
TraesCS7D02G535400	q7D-4	7D	627.32	*OsSBE1*	AK119436	Peng et al., 2014	1,4-alpha-glucan branching enzyme

^a^The candidate gene IDs correspond to the wheat IWGSC refseq v1.1 annotation. ^b^The reported gene names in rice. ^c^The homologous gene IDs in rice correspond to the rice IRGSP-1.0.39 annotation and GenBank database.

## Discussion

### Phenotyping of Wheat Quality Traits

Near-infrared reflectance spectroscopy (NIRS) has been widely used for quick, accurate, non-destructive, a highly repeatable assay of multiple quality traits in many crops (Delwiche et al., 1996; Windham et al., 1997; Tarr et al., 2012; Han et al., 2017; Xu et al., 2019). In wheat, it has been proved that reliable prediction of wheat composition is possible using NIR directly on the whole kernels, and especially for estimating essential grain components such as protein content and wet gluten content (Kuchel et al., 2006; Kristensen et al., 2018). Recently, NIRS has been used to evaluate the dough rheological properties in wheat (Jiang L. F. et al., 2019). In this study, traditional laboratory tests in some accessions were performed to determine the stability and reliability of NIRS quality characteristics. The correlation coefficients (R^2^) of the seven traits between NIRS estimated values and the results of conventional methods reached above 0.5 (**Supplementary Table S2**). Grain quality traits showed stronger correlations between NIRS estimates and actual values, such as WGC (0.75) and GTW (0.70), indicating that grain quality traits may be relatively more stable. Also, GWAS analysis using NIRS estimates as phenotypic data showed that at least half of the 40 core QTN regions were co-localized with QTLs and candidate genes previous identified using conventional methods (**Supplementary Table S6**). All of them confirmed that it was feasible to evaluate wheat quality traits by NIR spectroscopy and further to mine related candidate genes.

The dough rheological properties were often used as the key indicators to determine the strength of wheat gluten, which affected the processing quality of bread, steamed bun and noodles (Tsilo et al., 2013). In this study, the heritability of DST and DWA were lower except for DDT, which indicated that dough rheological properties were more susceptible to genotype **×** environment interactions (**Table 1**). Except for DDT-FY, DST-FY, and DWA-GPC, there were certain correlations between the dough rheological properties and grain quality traits. It is confirmed that processing quality was affected by multiple basic properties including GPC, WGC, TSC as well as other traits (Reese et al., 2007; Suchy et al., 2007).

In addition, it has been reported that plant height, spike length, peduncle length, flowering date and multiple yield traits affect these quality traits (Jiang J. et al., 2019). Based on our data for last few years, spikelet number, kernels per spike, thousand seed weight and grain yield were significantly negatively correlated with GPC, TSC and WGC, with the correlation coefficient ranged from −0.13 to −0.35 (unpublished data); plant height and peduncle length were significantly and positively correlated with GPC, TSC, WGC, SV, DWA and DDT, with the correlation coefficient ranged from 0.163 to 0.399 (unpublished data). In addition, flowering date was significantly negatively correlated with FY, due to the short grain filling time. All of these indicate that the improvement of dominant yield traits may reduce some quality traits, so the coordination of yield traits and quality traits is an important direction of wheat breeding.

### Comparison of GWAS Methods by Single-Locus and Multi-Locus

**Figure 7:**
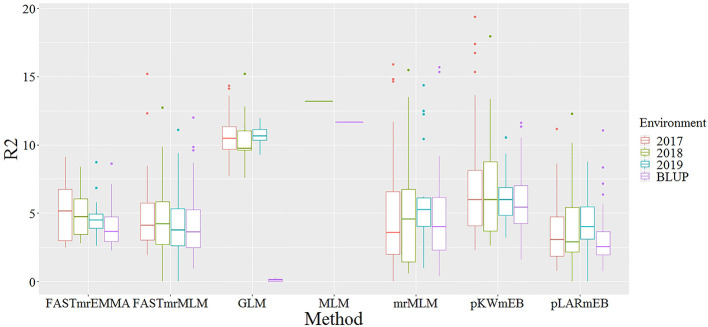
Comparison of interpretation rate of phenotypic variation for significant QTNs detected by two single-locus GWAS and five multi-locus GWAS methods in four environments. Different colored boxes represent different environments. The X axis represents different GWAS method, and the Y axis represents the interpretation rate of phenotypic variation (R^2^).

**Figure 8:**
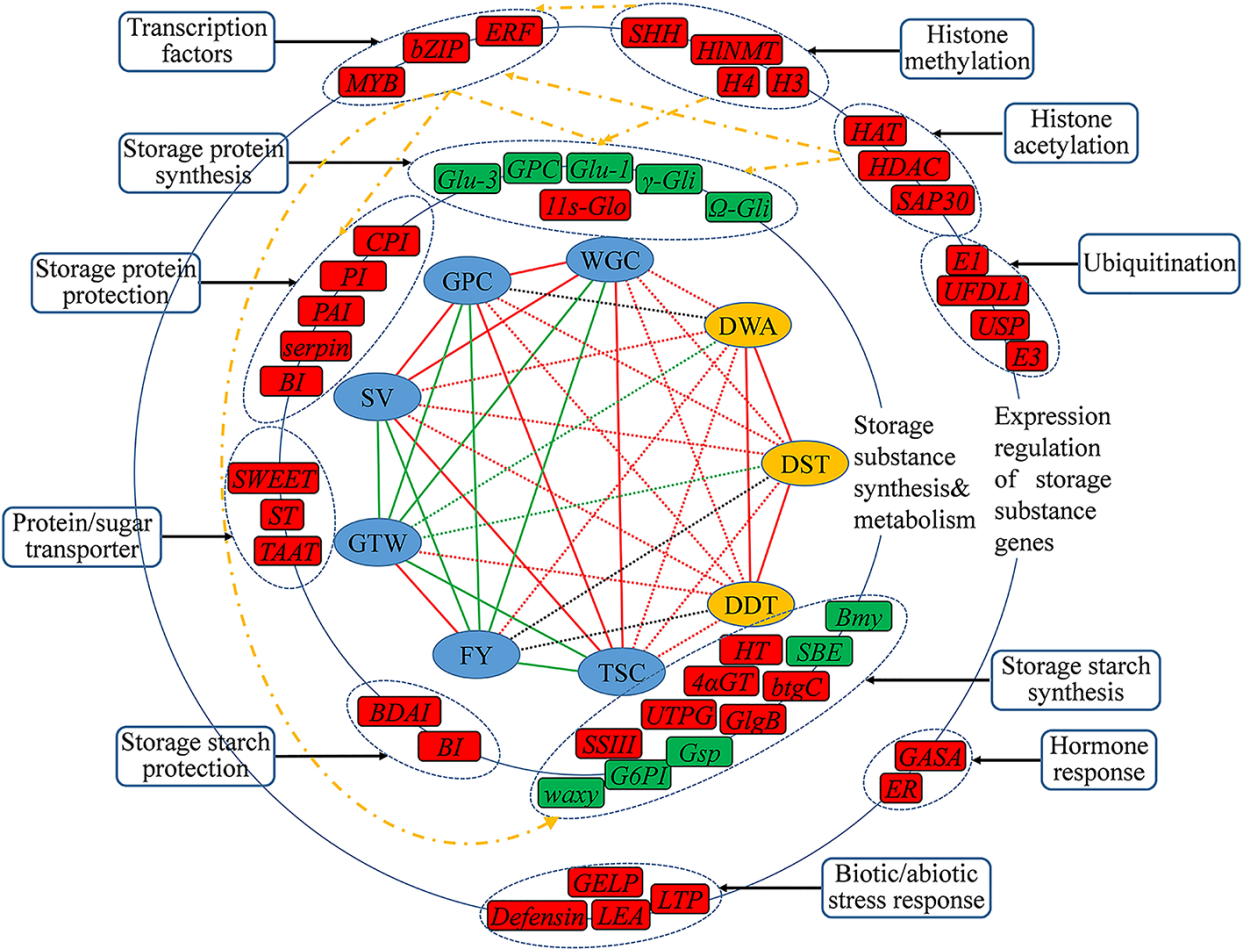
The inferred regulatory network of candidate genes that may regulating wheat quality traits. The innermost circle was the nine traits for GWAS analysis, of which the six grain quality traits in blue shade, and three dough rheological characteristics in yellow shade. The line linked the two traits represents their correlation level, with red line for a significant and positive correlation, and green line for a significant and negative correlation, and a dashed line for the correlation between the two types of traits. The middle and outer circles were the important candidate genes and their regulatory genes that may be involved in regulating the quality traits, respectively, with their functional categories indicated Genes in red shade are novel, in green shade are reported genes, and the yellow dotted lines represent possible regulatory networks.

With the rapid development of high-throughput sequencing and gene-chip technologies, GWAS has become a fast and simple method for analyzing the genetic variation of complex traits (Yang et al., 2014; Fan et al., 2016; Guo et al., 2017). It has been successfully applied in the studies of genetic variation of essential traits in multiple crops, including the grain yield in rice, male inflorescence size in maize, photosynthetic traits in soybean and free amino acid levels in wheat (Yang et al., 2014; Wu et al., 2016; Lü et al., 2018; Peng et al., 2018). Of these studies, some used the traditional single-locus GWAS methods, while others used the multi-locus methods. Due to the stricter Bonferroni correction, the single-locus GWAS methods, including MLM and GLM, were not efficient to detect small effective loci of a complex trait (Wang et al., 2016). Recently, GWAS analysis in many species using the multi-locus methods showed significantly higher efficiency in the detection of small effective loci than the single-locus methods (Hu et al., 2018; Lü et al., 2018; Peng et al., 2018). Excluding the FASTmrEMMA, the four other multi-locus GWAS approaches, which ranged from 89 to 118 and ranged from 2 to 81, found more QTNs than the two single-locus methods (**Table 3**). The multiple QTN loci identified by GLM method also showed weak consistency in different environments, and only a few QTNs could be detected simultaneously in two environments (**Supplementary Table S8**). In this study, the R^2^ values of significant QTNs detected by GLM were much higher than that by multi-locus GWAS methods (**Figure 7**), but not consistent, while, the R^2^ values of the QTNs detected by different multi-locus GWAS methods were relatively consistent and stable (**Figure 8**). Previous reports confirmed that the GLM model due to the absence of the Kinship matrix could generate some false-positive sites, accompanied by a shift in the phenotypic interpretation rate (Zhang et al., 2005; Yu et al., 2006). It indicated that multi-locus GWAS methods were more efficient than single-locus GWAS, especially on the validity and accuracy in detecting QTLs of complex traits controlled by multiple genes.

### QTN Regions for Wheat Quality Traits

In this study, more than half of the QTN regions (56%, 59/105) were correlated with grain quality traits and dough rheological properties, which was consistent with the significant correlation between the two types of traits (**Figure 2**). For the 40 core QTN regions, 14 were associated with at least two traits in two environments. Of which, seven regions were associated with grain quality traits and the remaining seven regions were correlated with both grain quality traits and dough rheological properties. Due to the high correlation of GPC, WGC and TSC, their QTLs were co-located in three core QTN regions, and three other regions were correlated with both GPC and WGC (**Table 4**). The pleiotropism of grain quality QTL was consistent with the previous reports, and breeding selections for these QTLs could simultaneously improve these quality traits (Li et al., 2013; Tsilo et al., 2013). Also, three regions were co-located with DDT and grain quality traits (including TSC, SV, WGC and GPC), and three regions were co-located with DWA and grain quality traits (including SV, FY and GTW). This was consistent with the extensive correlation between dough rheological properties and multiple grain quality traits, indicating that dough rheological properties were the result of the interaction of multiple basic grain quality traits.

We compared QTN regions detected in this study with the reliable QTLs in previous studies based on their physical locations of integrated genetic maps and significant association markers on chromosomes (**Supplementary Table S6**) and found that among the 40 core QTN regions, 21 were located on the same or close regions with previously reported QTLs (Kuchel et al., 2006; Mccartney et al., 2006; Li et al., 2013; Tsilo et al., 2013). More than half of the significant QTN regions were consistent with previous studies, confirming that association analysis based on NIR phenotyping and multi-locus GWAS methods was applicable in this kind of study. Overall, 4 (q1D-2, q2D-7, q6B-2 and q7A-7) of nine QTN regions stably detected in three environments related to grain quality traits were the same as previous studies (Kuchel et al., 2006; Li et al., 2013; Tsilo et al., 2013), while q7D-4 was previously reported as QTL for DDT of dough rheological properties (**Table 4** and **Supplementary Table S6**) (Li et al., 2013). For dough rheological properties, three (q1A-1, q2D-4 and q5D-1) of five QTN regions stably detected were consistent with previous reports (Li et al., 2013; Tsilo et al., 2013), while q3B-2 was previously reported as QTL for GPC of grain quality trait (Tsilo et al., 2013). It was also detected in a previous study (Kuchel et al., 2006) that q1A-2 was associated with both types of traits (FWA and DST). In addition, q7D-4 and q3B-2 were found to affect two types of quality traits, suggesting that these QTN regions could improve the processing quality of wheat grains in various aspects.

The QTN regions consistent with previous studies explained an average of 10.75% of phenotypic variation, while the new QTN regions explained only an average of 8.18%, confirming the advantage of the multi-locus GWAS methods in detecting small effective QTLs. Although these new QTN regions are non-primary, considering the extensive gene pleiotropy on quality traits, the exploration and utilization of these QTN regions cannot be ignored. Furthermore, the contribution of different genotypes to phenotypes using 16 SNP markers in 15 core QTN regions revealed, that eight SNP markers in eight regions had significant differences (at P <0.05) on the traits between the two contact genotypes in all four environments (**Figure 4**). These markers can be used as the priority loci for wheat quality improvement due to their higher contribution to phenotypes.

### Potential Candidate Genes and Possible Regulatory Networks for Wheat Quality Traits

Several studies have been performed on GPC and WGC, and some major genes that have a significant influence on GPC and WGC are reported as important traits affecting the final processing quality of wheat (Uauy et al., 2006; Sun et al., 2010; Conti et al., 2011; Li et al., 2012; Maphosa et al., 2013). Among them, the genes *GPC*, *Glu-1*, *Glu-3* and *Gli* encoding NAD transcription factor, HMW glutenin subunit, LMW glutenin subunit and gliadin, respectively, have been confirmed to be directly involved in the formation of grain protein, which has great impact on GPC and WGC (Uauy et al., 2006; Plessis et al., 2013). Here, multiple copies of these genes were found within or near the QTN regions in the present study, and all were highly expressed in grain endosperm, especially in the leaf late endosperm (**Figure 6** and **Supplementary Table S7**). The 11S seed storage globulin gene (*11S-Glo*) was also identified in the QTN region, and all of the above genes directly affected the synthesis of wheat grain storage protein.

Starch is another crucial substance for the wheat grain quality besides grain protein. Its composition and content can directly affect the flour gelatinization characteristics and determine the cooking quality (Toyokawa et al., 1989). The *waxy* gene encoding a granule-bound starch synthase I (GBSS I) to control the synthesis of amylose, having a significant impact on wheat starch quality (Nakamura et al., 1995), was found near the QTN region of q4A-3 in this study. Additionally, many genes encoding major enzymes associated with starch synthesis, such as *G6PI* (q1A-1, q1-D-3), *Gsp* (q5B-1), *SBE I* (q7A-8, q7D-4) and *BMY* (q4B-4, q5A-4) have also been identified in the QTN regions detected during the current study (**Supplementary Table S7**) (Wagner et al., 1996; Regina et al., 2005; Morris et al., 2013). Simultaneously, some genes in starch synthesis pathway were also identified in the candidate QTN regions, such as *SSIII* (q2A-1), *4αGT* (q2B-4), *HT* (q2D-9), *UTPG* (q2D-6), *GlgB* (q1A-3), *btgC* (q1D-4) and endoglucanase gene (q6A-1) (**Supplementary Table S7**). In general, most of the genes related to the formation of main storage protein and starch were detected in this study. These genes directly determine the protein and starch content, and their components, which were the direct control genes for the formation of protein or starch in grains.

In addition to finding the genes directly controlling the synthesis of storage protein and starch mentioned above, several genes related to the protection of storage substance were also identified in this study, such as *CPI* (q2D-10, q4B-4, q5A-4), *PAI* (q3A-6), *serpin* (q5A-2), *BDAI* (q3A-2, q3D-3) and *BI* (q7B-1) (**Supplementary Table S7**). These genes could prevent the hydrolysis of storage protein and starch, and their specific high expressions in endosperm at late grain development suggested that they played important roles in ensuring the normal synthesis and accumulation of storage substance (**Figure 6**) (Xiao and Li, 2004). Furthermore, some genes related to protein and sugar transport were also detected in the candidate QTN regions (**Supplementary Table S7**). Many studies confirmed that these genes had a great contribution to the accumulation and distribution of grain storage substance, so they were also essential for the formation of wheat quality (Peng et al., 2014; Sosso et al., 2015; Guo et al., 2018).

*AtZIP10*, *AtZIP25* and *AtZIP53* in *Arabidopsis* could regulate the expression level of storage protein genes by binding to their promoter region or interacting with other transcription factors, which were also reported in crops including maize, barley, rice, sorghum and wheat (Schmidt et al., 1992; Pirovano et al., 1994; Wu et al., 1998; Onate et al., 1999; Onodera et al., 2001; Lara et al., 2003; Alonso et al., 2009). A candidate gene encoding a bZIP transcription factor (*TraesCS6A02G096300*) specifically expressed in grain was found in q6A-1 (**Supplementary Table S7**). Simultaneously, MYB transcription factors have been confirmed to be involved in the accumulation of storage substance by regulating the expression levels of GA-response and storage substance genes (Gubler et al., 1995; Suzuki et al., 1998; Diaz et al., 2002; Plessis et al., 2013). Several candidate genes encoding MYB transcription factors were detected in this study, and all expressed specifically in grains, which could be considered as important candidate genes for wheat quality improvement (**Figure 6** and **Supplementary Table S7**). Furthermore, two ethylene-responsive transcription factor genes with specific high expression in grains have also been identified, as ethylene has a strong effect on grain development. In summary, several transcription factor genes were detected in the candidate QTN regions, and all had high expression levels in the prophase and anaphase of endosperm development, which suggested that they may play regulatory roles in the synthesis and accumulation of grain storage substance.

Recently, the regulation of seed development by epigenetic levels, especially the expression regulation of grain storage proteins, has been reported in many species (Gehring et al., 2009; Hsieh et al., 2009; Locatelli et al., 2009; Zemach et al., 2010; Wen et al., 2012). DNA methylation modification mainly regulated the expression level of gliadin genes in grains by regulating the methylation levels of multi-region of *Gli* genes, thereby affecting the grain protein content (Sturaro and Viotti, 2001; Locatelli et al., 2009; Zemach et al., 2010; Wen et al., 2012). In addition, the role of histone acetylation/deacetylation in seed development has also been reported in many species (Tanaka et al., 2008; Fu et al., 2010; Yang et al., 2015). The expression level of *Arah3* encoding a storage protein in peanut seeds was similar to its acetylation level, while histone deacetylase genes *AtHDA19* and *AtHDA6* in Arabidopsis affected seed storage protein expression by participating in the binding of transcription factors with the promoter regions of the target genes (storage protein genes) (Tanaka et al., 2008). In this study, a large number of genes in detected QTN regions involved in DNA methylation and histone acetylation/deacetylation were specifically expressed in grains (**Supplementary Table S7**). It is worth noting that *HDAC* (*TraesCS1A02G317100*) found in a QTN region (q1A-4) was specifically expressed in grain, suggesting that it may have similar functions as *AtHDA19* and *AtHDA6*. Furthermore, multiple ubiquitination-related genes specifically expressed in grains were found in the detected QTN regions (**Supplementary Table S7**), which may also be associated with the development of grain. In summary, multiple grain-expressed genes related to histone modification including DNA methylation, histone acetylation and histone ubiquitination were identified in detected QTN regions, which suggested that histone modification in various forms might be widely involved in the development and quality formation of wheat grain.

Finally, benefit from the high detection rate of multi-locus GWAS methods for small effective QTLs, based on the functional annotation of candidate genes, tissue expression characteristics and previous studies, we preliminarily drew the regulatory network of genes that may involve in the formation of wheat grain quality (**Figure 8**). Among all these candidate genes, the genes involved in the biosynthesis and metabolism of storage substance were considered to be the first level affecting the formation of wheat grain quality, including storage protein synthesis, starch synthesis, storage protein protection, starch protection and protein/sugar transport, and these genes could directly control the synthesis and accumulation of storage substance in grains. The second level mainly included transcription factor, histone modification, stress response and hormone response genes, which affected the wheat grain quality through indirect pathways. These identified transcription factors could affect the formation of grain quality by binding to the promoter regions of the first level genes, and histone modification could occur on the second level transcription factor genes or directly affect the first level genes. From our results, the development and quality formation of wheat grain showed a complex network regulation pattern, which was the result of the combined actions of multi-level gene regulations.

## Conclusion

In this study, two single-locus and five multi-locus GWAS analysis were performed for six grain quality traits and three dough rheological properties based on 19,254 SNPs in 267 wheat accessions. 299 QTNs within 105 regions were identified to be associated with these quality traits in four environments. Among 105 QTN regions, 40 core QTN regions were stably detected in at least three environments, 19 of which are novel. After a detailed function analysis, 67 core candidate genes were determined to be related to grain quality formation. Finally, based on the previous knowledge and results in this study, a preliminary regulatory network of genes may involve in wheat quality formation was established. This study verified the power and reliability of multi-locus GWAS methods in wheat quality trait study, and increased the understanding of wheat quality formation mechanisms. The detected QTN regions and candidate genes could be further used for characterization of genes regulating wheat quality and marker-assisted breeding for improving grain quality in wheat.

## Data Availability Statement

The raw data supporting the conclusions of this article will be made available by the authors, without undue reservation.

## Author Contributions

YY and YC performed the experiment and wrote the paper. XZ, DW, and ZZ participated in the field trials and trait evaluation. DW conducted the laboratory trait measurements. Y-GH and LC designed the experiment and evaluated the paper. All authors contributed to the article and approved the submitted version.

## Funding

This work was funded by the National Natural Science Foundation of China (Grant No. 31671695; No. 31501307), the sub-project of the 863 Program (2013AA102902) of the Ministry of Sci-Tech, the China 111 Project (B12007) of the Ministry of Education of China.

## Conflict of Interest

The authors declare that the research was conducted in the absence of any commercial or financial relationships that could be construed as a potential conflict of interest.
