# Supplementary figures and images for: Multi-Locus GWAS of Quality Traits in Bread Wheat: Mining More Candidate Genes and Possible Regulatory Network
Source: Front Plant Sci. 2020 Jul 31;11:1091. doi: 10.3389/fpls.2020.01091 (PMC7411135; doi:10.3389/fpls.2020.01091)

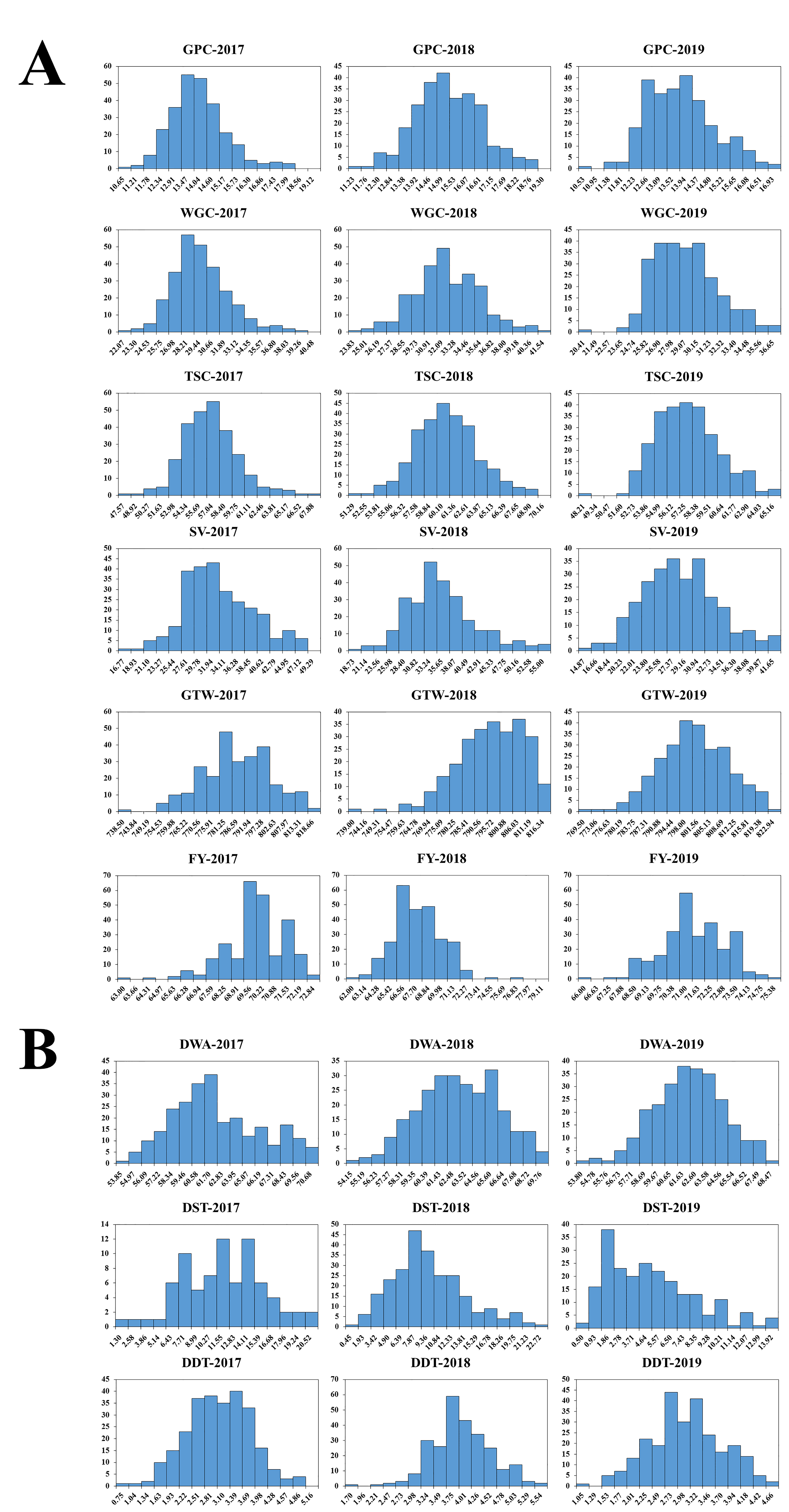

Supplement: Supplementary file 2 [file Image_1.tif]

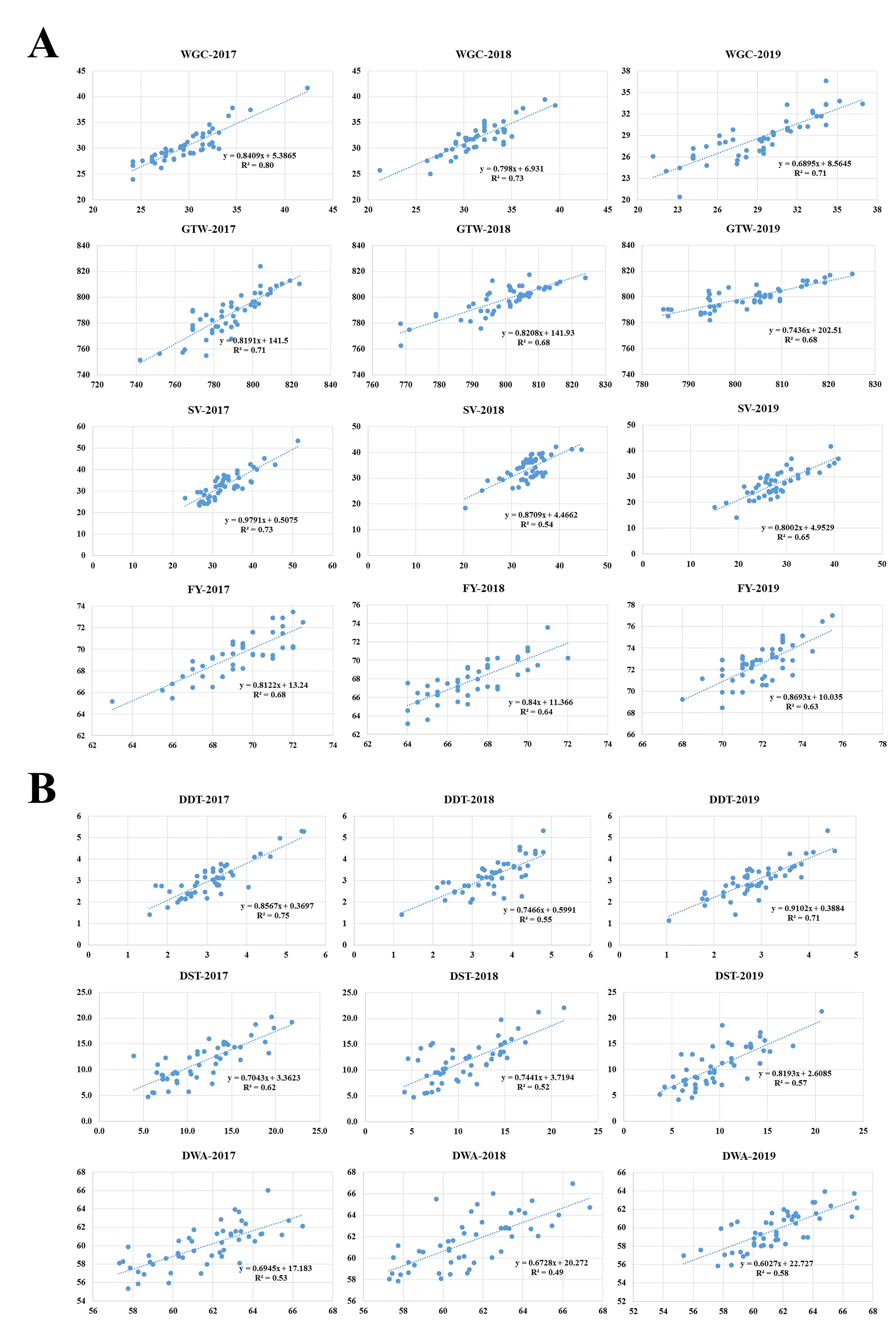

Supplement: Supplementary file 3 [file Image_2.tif]
